# Upregulated GSDMB in Clear Cell Renal Cell Carcinoma Is Associated with Immune Infiltrates and Poor Prognosis

**DOI:** 10.1155/2021/7753553

**Published:** 2021-12-16

**Authors:** Yuanshan Cui, Zhongbao Zhou, Yumeng Chai, Yong Zhang

**Affiliations:** ^1^Department of Urology, Beijing Tian Tan Hospital, Capital Medical University, Beijing, China; ^2^Department of Urology, The Affiliated Yantai Yuhuangding Hospital of Qingdao University, China

## Abstract

Gasdermin B (GSDMB) is part of the gasdermin (GSDM) family, and they use varying means of domain interactions in molecules to adjust their pore-forming and lipid-binding actions. The GSDM family has roles in the regulation of cell differentiation and proliferation, particularly in the process of pyroptosis. Nonetheless, the correlation of GSDMB with immune infiltrates and its prognostic values in clear cell renal cell carcinoma (ccRCC) are still undefined. Therefore, we assessed the correlation of GSDMB with immune infiltrates and its prognostic role in ccRCC. The transcriptional expression profiles of GSDMB in ccRCC tissues in addition to normal tissues were retrieved from The Cancer Genome Atlas (TCGA) and additionally verified in a different independent cohort, which was obtained from the Gene Expression Omnibus (GEO) database. The Human Protein Atlas and the Clinical Proteomic Tumor Analysis Consortium (CPTAC) were used to assess the protein expression of GSDMB. To assess the effectiveness of GSDMB in distinguishing ccRCC from normal samples, the receiver operating characteristic (ROC) curve analysis was performed. Relationships between GSDMB expression, clinicopathological variables, and overall survival (OS) were evaluated with multivariate methods as well as Kaplan-Meier survival curves. Protein-protein interaction (PPI) networks were created with STRING. Functional enrichment analyses were conducted by utilizing the “ClusterProfiler” package. The Tumor Immune Estimation Resource (TIMER) and tumor-immune system interaction database (TISIDB) were utilized to determine the association between the mRNA expression of GSDMB and immune infiltrates. GSDMB expression was significantly more upregulated in ccRCC tissues compared to surrounding normal tissues. An increase in the mRNA expression of GSDMB was related to the high pathologic stage and advanced TNM stage. The analysis of the ROC curve indicated that GSDMB had an AUC value of 0.820 to distinguish between ccRCC tissues and adjacent normal controls. Kaplan-Meier survival analysis indicated that ccRCC patients with high GSDMB had a poorer prognosis compared to those with low GSDMB (*P* < 0.001). Correlation analysis showed that the mRNA expression of GSDMB was associated with immune infiltrates and the purity of the tumor. Upregulation of GSDMB is significantly related to immune infiltrates and poor survival in ccRCC. The results of this study indicate that GSDMB could be regarded as a biomarker for the detection of poor prognosis and potential target of immune treatment in ccRCC.

## 1. Introduction

Renal cell carcinoma's (RCC) incidence has been growing on a global scale in the last few decenniums, and RCC has the highest mortality rate annually among urological carcinomas [1]. RCC is a heterogeneous type of carcinoma, of which the most common form is clear cell RCC (ccRCC) which makes up 75-80% of RCCs [2]. Due to the resistance to chemotherapy and radiotherapy, the current treatment of ccRCC patients is still unsatisfactory. Therefore, resecting the tumor is the most optimal choice as treatment of ccRCC patients, which is regarded as the sole type of treatment that could lead to complete curation [3]. Generally, the majority of ccRCC patients are diagnosed in an advanced stage, as a result of an occult onset and rapid progression [4]. Although targeted therapy has shown a positive effect on extending the duration of patients' survival time, the drug resistance associated with long-term use was still a problem that has not been settled [5]. Immune therapy, in particular immune checkpoint inhibitors, is a type of treatment for ccRCC patients that is very promising [6]. However, not every patient can benefit from it since research has shown that the objective response rate to anti PD-L1 therapy is merely 20% approximately. The patients who did have a positive response to immune checkpoint inhibitors did not exhibit long-term remission [7]. The proliferation mechanism of ccRCC has a complex and multifactorial nature, consisting of an elaborate network of different genetic backgrounds and multiple carcinogens that result in changes in oncogenes or tumor suppressors [8]. Thus, it is a necessity to determine the molecular mechanisms that are related to the progression of ccRCC, which is valuable for diagnosis and treatment.

A new kind of programmed cell death known as pyroptosis has vital functions in both immune defense and septic shock [9]. It is also known as programmed cell death mediated by gasdermin. It is known that the gasdermin (GSDM) family has different functions in the regulation of both cell proliferation and differentiation containing GSDMA, GSDMB, GSDMC, GSDMD, GSDME, and DFNB59 [10]. GSDMB and GSDMA genes are found in chromosome 17q2, and GSDMC and GSDMD can be found in chromosome 8q24 [10]. Except for DFNB59, the other family members of the gene share an approximate 45% of sequence homology; in addition, each GSDM has two domains that can bind one another and are attached via a long type of flexible linker [11]. With the exception of DFNB59, other known members of the GSDM family have comparable 3D structures as indicated by the sequence homology [12]. The gasdermin-N domain allows the majority of GSDM members to serve as a novel kind of pore-forming protein. While they are executing their function as pore-forming proteins, multiple GSDM family members may use varying processes of interactions between intramolecular domains that modify their pore-forming and lipid-binding actions, possibly inducing pyroptosis-like qualities in these cells. In GSDMB, one of the members of the GSDM family, pyroptosis-like features have also been observed, and several studies have suggested that overexpression of GSDMB exists in multiple types of carcinomas, in which it could be correlated with the progression of cancer and metastasis. However, the value of GSDMB in prognosis and its relation with immune infiltrates in ccRCC are yet to be completely elucidated.

In this article, we downloaded data and evaluated the association between GSDMB expression, clinical data, and overall survival (OS) in patients with ccRCC by using the different databases TCGA, GEO, and Human Protein Atlas. Then, the TIMER and GEPIA databases were used in order to identify the correlation between GSDMB expression and immune cells that have infiltrated and their equivalent sets of gene markers. Besides, we used the STRING website to explore the GSDMB-interacted protein network. Results demonstrated that high GSDMB level was correlated with poor prognosis and related to an inadequate infiltration of immune cells in ccRCC. Hence, there is a strong possibility that GSDMB overexpression may undermine the antitumor effects of the immune system in ccRCC.

## 2. Method

### 2.1. The Cancer Genome Atlas (TCGA) and the Gene Expression Omnibus (GEO) Database

TCGA database (https://genome-cancer.ucsc.edu/) is a freely accessible data portal of a large-scale project on cancer genomes that consists of pathological as well as clinical data of over 30 different kinds of cancer and is intended for research. TCGA tool cancer browser was used to obtain data of ccRCC patients, including RNA-Seq expression and corresponding data on clinical pathology. The GEO database, an extensive gene expression library, can be found in the National Center of Biotechnology Information (NCBI) (https://www.ncbi.nlm.nih.gov/geo/). Since this database is available to the public by means of open access, authorization from the local ethics committee was not necessary.

### 2.2. The Human Protein Atlas (HPA), UALCAN, and Clinical Proteomic Tumor Analysis Consortium (CPTAC)

The HPA consists of a large amount of information on proteomics and transcription of individual human samples containing tissue, cell, and a pathology atlas. The online database contains information of cell-specific locations for over 40 different healthy tissues as well as 20 most common categories of carcinomas. Furthermore, data on protein immunohistochemistry in human tumor tissues and normal tissues is also available on the HPA website. UALCAN (http://ualcan.path.uab.edu/) is a convenient and simple-to-use online resource that can be used for analyzing publicly available data on cancer. Using proteomics technologies, CPTAC (http://ualcan.path.uab.edu/analysis-prot.html) evaluates tumor biospecimens by mass spectrometry, which identifies and quantifies the characterizing proteome and constituent proteins of every tumor sample. In the present report, we used UALCAN to perform a throughput analysis of GSDMB protein expression obtained from CPTAC.

### 2.3. Univariate and Multivariate Logistic Regression Analyses

In order to identify the impact of the expression of GSDMB in ccRCC patients, univariate Cox regression analysis was conducted to calculate the relation between GSDMB's expression level and OS of patients across two different cohorts. Then, multivariate analysis was conducted to evaluate whether GSDMB is a distinct prognostic factor of survival in ccRCC patients. GSDMB was considered statistically significant in the Cox regression analysis when *P* < 0.05.

### 2.4. Protein-Protein Interaction (PPI) Networks and Functional Enrichment Analysis

The Search Tool for the Retrieval of Interacting Genes/Proteins (STRING) website (https://string-db.org/) is another online tool. On this website, a large collection of integrating and consolidated PPI data is hosted. The PPI network information could be obtained after importing the GSDMB into STRING. A confidence score of >0.7 was regarded as significant. The “ClusterProfiler” package was used to perform Gene Ontology (GO) enrichment as well as Kyoto Encyclopedia of Genes and Genomes (KEGG) pathway analyses of coexpression genes, which were visualized with the “ggplot2” package [13].

### 2.5. Tumor Immune Estimation Resource (TIMER) Database

TIMER (https://cistrome.shinyapps.io/timer/) is an extensive web-based resource that can be used for systematic analysis of immune infiltrates in various kinds of cancers. In the present study, we applied TIMER to establish the association among GSDMB's expression in ccRCC and six different types of immune infiltrates (B cells, CD4-positive T cells, CD8-positive T cells, macrophages, neutrophils, and dendritic cells).

### 2.6. The Gene Expression Profiling Interactive Analysis (GEPIA) Analysis

The GEPIA (http://gepia.cancer-pku.cn/index.html) is a database, which can be accessed online and is comprised of 8587 normal and 9736 tumors samples from GTEx and TCGA data. The database is dedicated to different types of analyses regarding the expression of RNA sequencing. We used it to analyze the association among GSDMB expression and various immune cell markers. The *x*-axis in the graph represented the amount of GSDMB expression, while the *y*-axis represented other type of genes of interest. Furthermore, TIMER data was used to verify which genes had a significant association with GSDMB expression as indicated by the GEPIA website.

### 2.7. Tumor-Immune System Interaction Database (TISIDB)

TISIDB (http://cis.hku.hk/TISIDB/) is an integrated repository web portal, accessible online, for information on the correlation that exists between tumors and the innate immune system. In this article, we used the TISIDB to establish GSDMB expression and tumor-infiltrating lymphocytes (TILs) in cancers occurring in Homo sapiens. The relative abundance of TILs was deduced from the gene expression profile, which was derived through gene set variation analysis. Spearman's test was conducted to quantify the associations between GSDMB and TILs.

### 2.8. Statistical Analyses

All of the statistical analyses were conducted with R (V 3.6.3), and the R package ggplot2 was utilized to observe the differences in expression. The Mann-Whitney *U* test and paired *t*-test were conducted to establish the differences among ccRCC tissues and surrounding normal tissues. The pROC package was used to visualize the ROC curve, in which the cutoff value of GSDMB could be detected. To evaluate the effect of GSDMB on survival, log-rank and Kaplan-Meier tests were performed by using the survminer package. Correlation analysis was used by the Pearson correlation and Spearman test.

## 3. Result

### 3.1. Expression Pattern of GSDMB in Pan-Cancer Perspective

**Figure 1:**
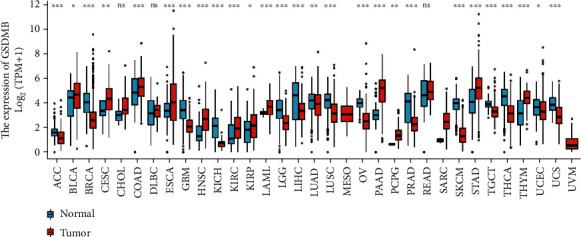
Expression pattern of GSDMB in a pan-cancer perspective. The mRNA expression of GSDMB was upregulated in 12 of 33 cancer types and downregulated in 15 of all 33 cancer types compared with normal tissues (ns: *P* ≥ 0.05; ∗: *P* < 0.05; ∗∗: *P* < 0.01; ∗∗∗: *P* < 0.001).

The complete working set contained 33 types of cancer of which the mRNA expression pattern of GSDMB was evaluated. As shown in Figure 1, in comparison to normal tissues, GSDMB was significantly upregulated in 12 out of 33 cancer types and downregulated in 15 out of 33 cancer types. The data demonstrated that the mRNA expression of GSDMB was expressed in an abnormal way throughout different types of cancers.

### 3.2. Upregulated mRNA and Protein Expression of GSDMB in ccRCC Patients

**Figure 2:**
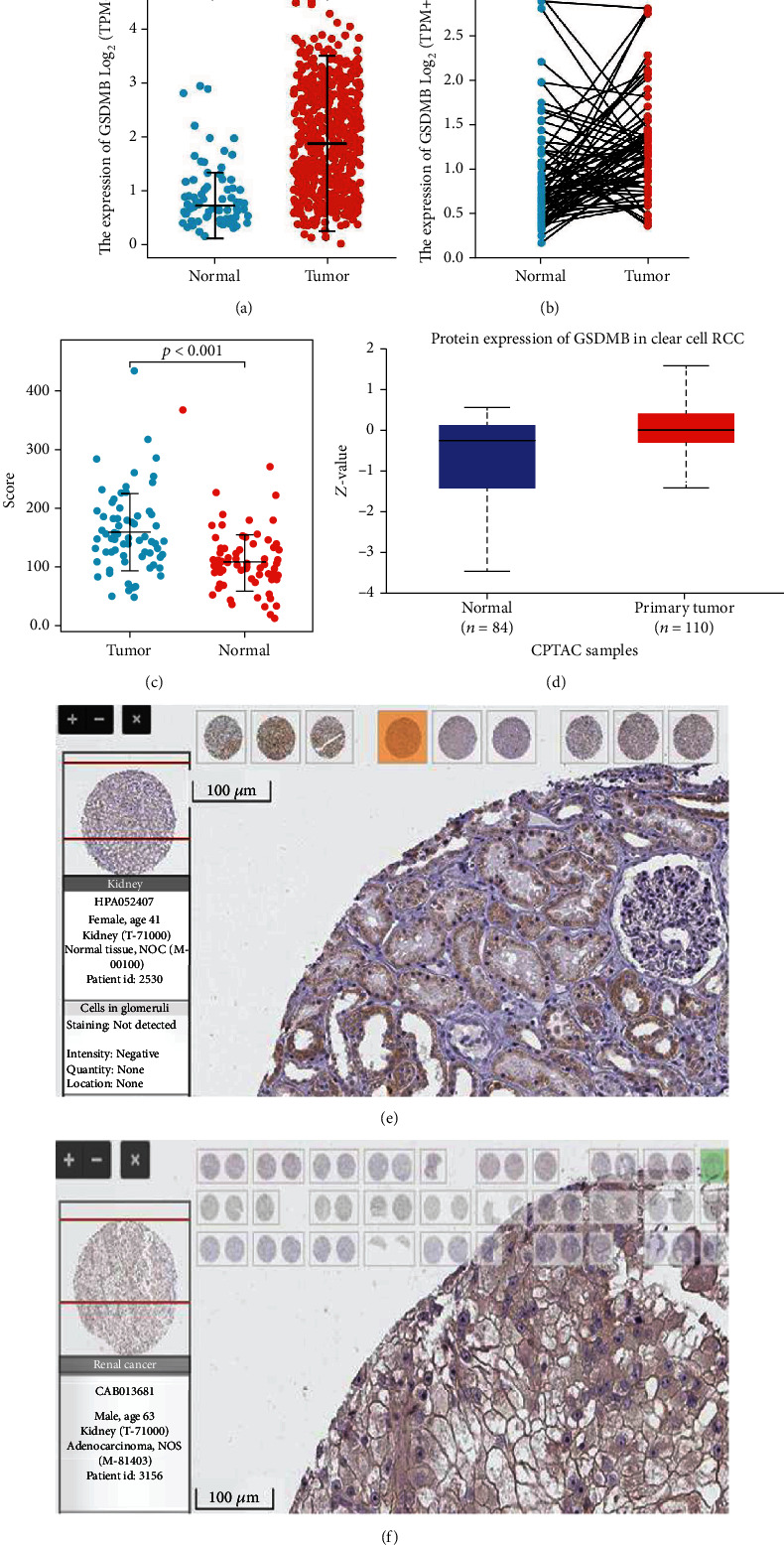
The mRNA and protein expression of GSDMB in ccRCC. (a) mRNA expression levels of GSDMB in 539 ccRCC samples and 72 normal samples. (b) mRNA expression levels of GSDMB in 72 ccRCC and matched-adjacent normal samples. (c) mRNA expression levels of GSDMB in 72 ccRCC and matched-adjacent normal samples in the GEO database. (d) Protein expression levels of GSDMB based on CPTAC. (e) Normal tissues: the protein levels of GSDMB based on the Human Protein Atlas. (f) Tumor tissues: the protein levels of GSDMB based on the Human Protein Atlas (^∗∗∗^*P* < 0.001). ccRCC: clear cell renal cell carcinoma.

In order to establish the mRNA as well as protein expression of GSDMB in ccRCC, data on GSDMB expression found in TCGA, GEO, and HPA were analyzed.  Figure 2(a) shows the unpaired data analysis indicating that the levels of mRNA expression of GSDMB in ccRCC (*n* = 539) were significantly greater compared to those in surrounding normal tissues (*n* = 72) (1.93 ± 0.968 vs. 0.895 ± 0.613, *P* < 0.001). Subsequent paired data analyses demonstrated that the levels of mRNA expression of GSDMB in ccRCC tissues (*n* = 72) were significantly greater in comparison to those in surrounding healthy tissues (*n* = 72) (Figure 2(b), 1.238 ± 0.585 vs. 0.895 ± 0.613, *P* < 0.001), which was also validated in the GEO database (GSE53757) (Figure 2(c), 159.474 ± 66.165 vs. 107.306 ± 47.003, *P* < 0.001) (Figure 2(c)). We conducted analysis on CPTAC via UALCAN to show throughput analysis of GSDMB protein expression. The results indicated that the protein expression of GSDMB in ccRCC (*n* = 110) was significantly greater compared to that in healthy tissues (*n* = 84) (Figure 2(d)). As shown in Figures 2(e) and 2(f), immunohistochemical staining from HPA demonstrated that GSDMB protein was also upregulated in ccRCC tissue. These findings suggest that the mRNA as well as protein expression of GSDMB is upregulated in ccRCC.

### 3.3. Relationships between GSDMB mRNA Levels and Clinical Pathological Features of ccRCC Patients

**Figure 3:**
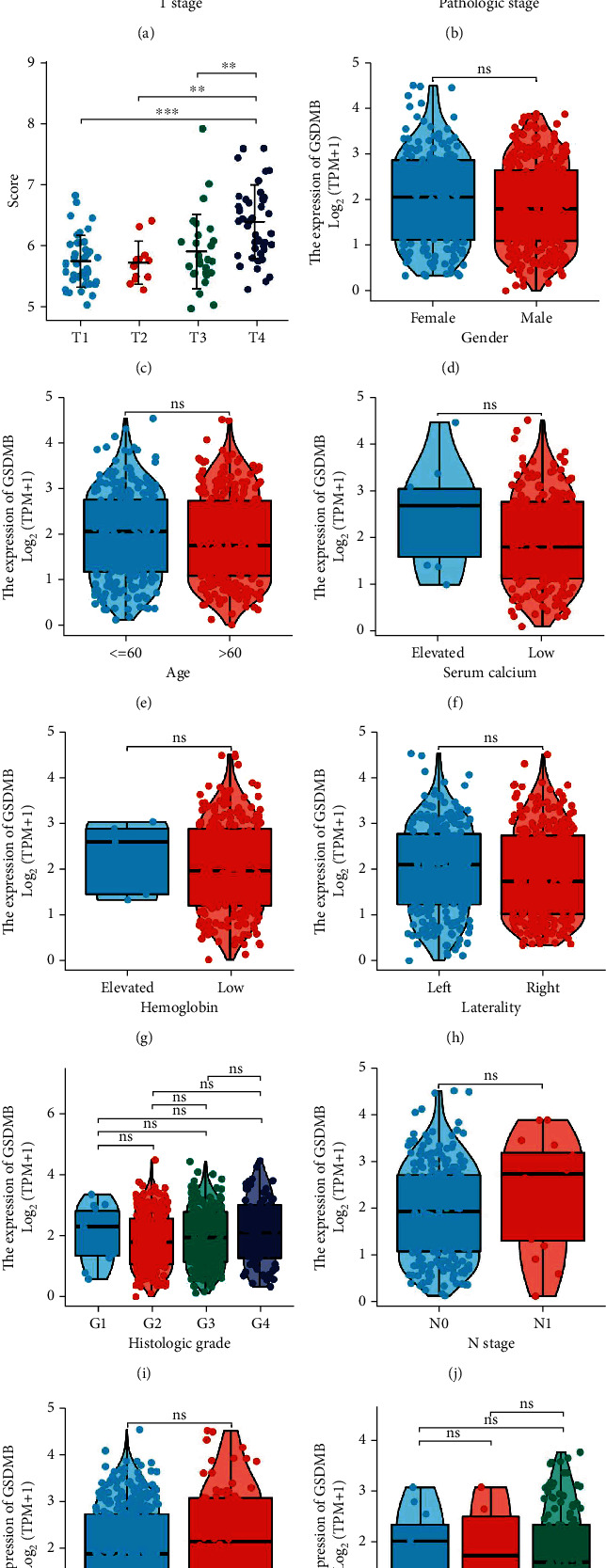
Relationships between GSDMB mRNA levels and clinical pathological characteristics. Higher expression levels of GSDMB were observed in patients with a high T stage (a, c) and patients with a high pathologic stage (b). No statistically significant correlation was found between the expression levels of GSDMB and gender (d), age (e), serum calcium (f), hemoglobin (g), laterality (right or left) (h), histologic grade (i), N stage (j), M stage (k), or primary therapy outcome (l) (ns: no significance; ^∗^*P* < 0.05, ^∗∗^*P* < 0.01, and ^∗∗∗^*P* < 0.001).

Dunn's test and the Kruskal-Wallis test were conducted to assess the relation among GSDMB mRNA expression and clinical pathological features of ccRCC samples.  Table 1 shows the baseline features of ccRCC patients which were retrieved after accessing TCGA database. As shown in Figures 3(a)–3(l), higher levels of GSDMB expression were identified in patients with a high T stage (Figure 3(a)) and patients with a high pathologic stage (Figure 3(b)). Besides, the GEO database also demonstrated that GSDMB was upregulated in patients with a high T stage (Figure 3(c)). Nonetheless, statistically significant differences were not observed among the levels of GSDMB expression and diverse clinical pathological features, including gender (Figure 3(d)), age (Figure 3(e)), serum calcium (Figure 3(f)), hemoglobin (Figure 3(g)), laterality (right or left) (Figure 3(h)), histologic grade (Figure 3(i)), N stage (Figure 3(j)), M stage (Figure 3(k)), or primary therapy outcome (Figure 3(l)). Overall, these outcomes suggested that GSDMB is associated with the high T stage, which additionally suggests that GSDMB may have a role as a biomarker of poor prognosis in ccRCC.

### 3.4. Differential RNA-Seq Levels of GSDMB as a Potential Biomarker to Differentiate between ccRCC and Normal Samples

**Figure 4:**
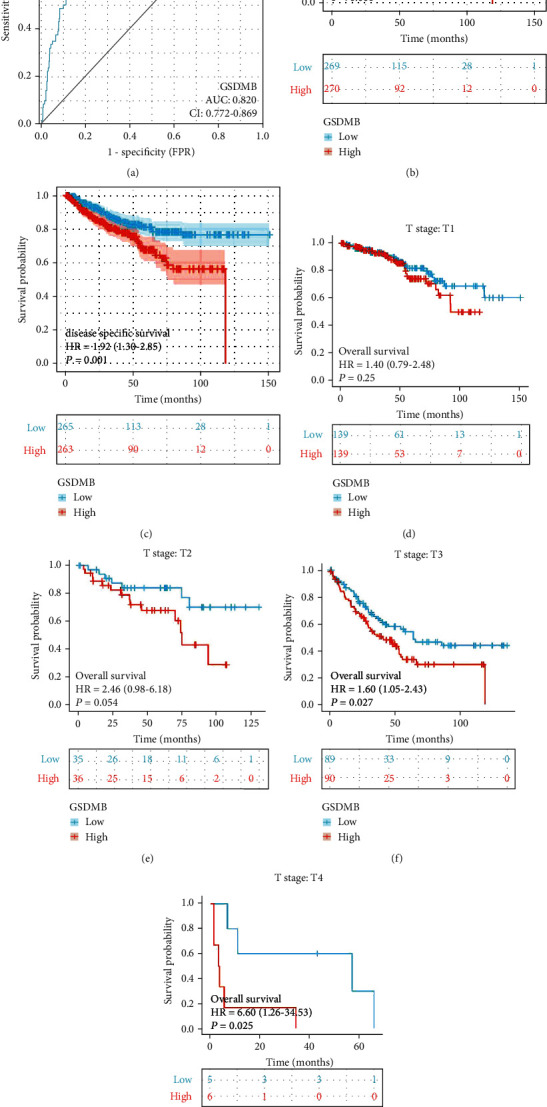
ROC and Kaplan-Meier curves for GSDMB. (a) ROC curve showed that GSDMB had an AUC value of 0.820 to discriminate ccRCC tissues from healthy controls. (b, c) Kaplan-Meier survival curves indicated that ccRCC patients with high GSDMB mRNA expression had a shorter OS and DSS than those with low-level of GSDMB. Besides, a subgroup analysis was performed on T1, T2, T3, and T4, respectively (d–g). It showed that GSDMB correlates with high T stage and unfavourable prognosis. OS: overall survival; DSS: disease-specific disease.

To assess the effectiveness of GSDMB in distinguishing ccRCC from normal samples, the ROC curve analysis was performed. The ROC curve analysis in Figure 4(a) demonstrated that GSDMB was associated with an AUC value of 0.820 (95% CI: 0.772–0.869). Based on a cutoff value of 1.062, GSDMB showed a sensitivity, specificity, and accuracy of 75.0, 77.2, and 76.9%, respectively. Furthermore, the positive predictive value was 30.5% while the negative predictive value was 95.9%. In univariate analysis, T stage (*P* < 0.001), N stage (*P* < 0.001), M stage (*P* < 0.001), pathologic stage (*P* < 0.001), age > 60 (*P* < 0.001), histologic grade (*P* < 0.001), and GSDMB expression (*P* < 0.001) were associated with OS. In multivariate analysis, only M stage (*P* < 0.001), age > 60 (*P* = 0.009), histologic grade (*P* = 0.049), and GSDMB (*P* = 0.002) expression were independent prognostic factors for ccRCC (Table 2). These results showed that GSDMB may be a valuable biomarker for the differentiation between ccRCC and normal tissues.

### 3.5. High mRNA Expression of GSDMB Is Correlated with Poor OS and Disease-Specific Survival (DSS)

Kaplan-Meier curves were carried out to examine the correlation between mRNA expression of GSDMB and OS and DSS in ccRCC patients. Figures 4(b) and 4(c) show that the OS and DSS of ccRCC patients that had a high level of GSDMB were significantly shorter compared to those of a low level of GSDMB (hazard ratio (HR) = 1.98 (1.45-2.71), *P* < 0.001; HR = 1.92 (1.30-2.85), *P* = 0.001). Besides, a subgroup analysis was performed on T1, T2, T3, and T4, respectively (Figures 4(d)–4(g)). It showed that GSDMB correlates with high T stage and unfavourable prognosis. Taken together, these results demonstrated that an elevated mRNA expression of GSDMB may have a role as a biomarker associated with poor prognosis in ccRCC.

### 3.6. Increased Expressions of GSDMB Associated with Poor Prognosis in Various Stages of Cancer

**Figure 5:**
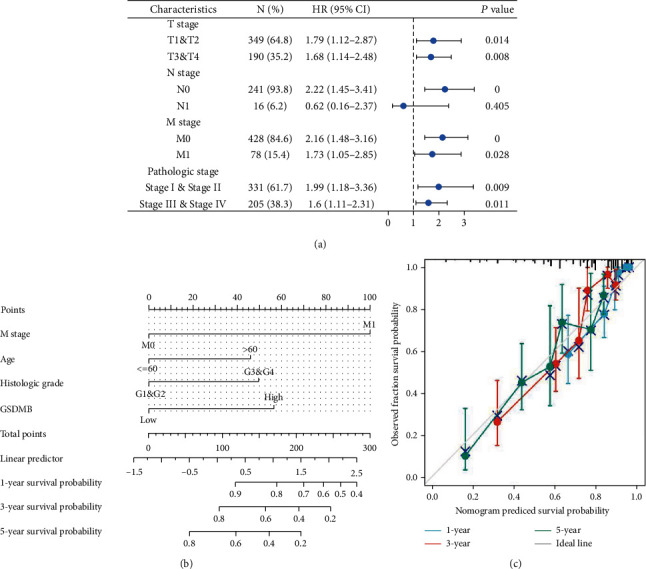
(a) Multivariate survival analysis of overall survival probabilities concerning GSDMB expression in patients with different subgroups according to the cancer stage. (b) Nomogram for predicting the probability of 1-, 3-, and 5-year OS for ccRCC patients. (c) Calibration plot of the nomogram for predicting the OS likelihood. ccRCC: clear cell renal cell carcinoma; OS: overall survival.

The results of the Kaplan-Meier survival analysis indicated that ccRCC patients with a high level of GSDMB expression were associated with a poorer prognosis in comparison to patients with a low level of GSDMB expression in the following categories of various stages of cancer: T (T1 and T2, *P* = 0.014; T3 and T4, *P* = 0.008), N (N0, *P* = 0, N1; *P* = 0.405), M (M0, *P* = 0; M1, *P* = 0.028), and pathologic stage (I and II; *P* = 0.009; III and IV, *P* = 0.011) (Figure 5(a)). These findings indicate that the GSDMB's expression level can influence the prognosis of ccRCC patients in various pathological stages.

### 3.7. Construction and Verification of a Nomogram on the Basis of GSDMB Expression

In order to present a useful quantitative model that can assist clinicians in establishing the correct prognosis of ccRCC patients, we constructed a nomogram which combined the clinical features of patients that were independently correlated with survival through multivariate analysis (M stage, age, histologic grade, and GSDMB; Figure 5(b)). A point scale was used to appoint the locations of these variables in the nomogram according to the multivariate Cox analysis as follows: we used a straight line to identify the number of points for the variables in the nomogram, and the total number of the points appointed to every variable was rescaled on a scope between 0 and 100. The different locations of the variables were summed and then listed as the total number of points. Vertical lines were drawn from the axis of total points downward to the outcome axis to identify the expected survival of ccRCC patients after 1, 3, and 5 years. The C-index of the nomogram was 0.774 with 1000 bootstrap replicates. The bias-corrected line, which was visualized in the calibration plot, was nearing the ideal curve (also referred to as the 45-degree line), which represents a fair agreement between the observed and predicted values (Figure 5(c)). Taken together, the results have shown that the nomogram is a superior model capable of establishing long-term survival (1, 3, and 5 years) in ccRCC patients than individual prognostic factors.

### 3.8. Identifying DEGs in High and Low GSDMB Expression Groups

**Figure 6:**
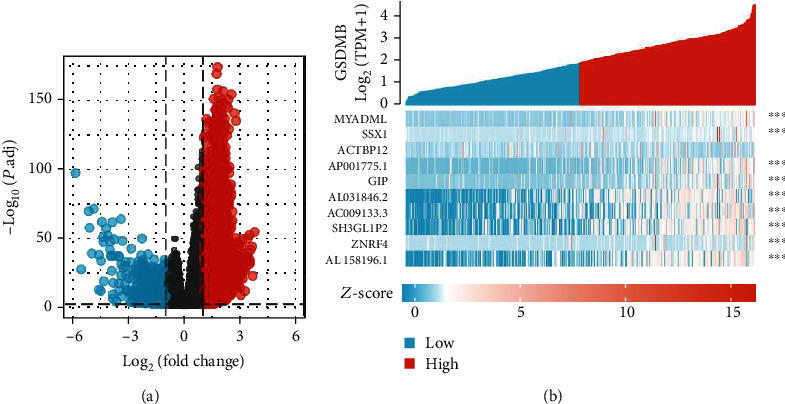
Differentially expressed genes between patients with high and low GSDMB expression. (a) Volcano plot of differentially expressed genes between the high and low GSDMB expression groups. Normalized expression levels are shown in descending order from green to red. (b) Heatmap of the top ten significant differentially expressed genes between the high and low GSDMB expression groups. Green and red dots represent downregulated and upregulated genes, respectively.

The DSEeq2 package in R (∣logFC | >2, modified *P* value <0.05) was used to analyze the data from TCGA, and 1331 DEGs were detected in the high level of the GSDMB expression group and low level of the GSDMB expression group; among these, 1197 were upregulated and 134 downregulated genes in the high expression group (Figure 6(a)).  Figure 6(b) shows the heatmap of the ten most significant DEGs in the high-level and low-level GSDMB expression groups.

### 3.9. PPI Networks and Functional Annotations

**Figure 7:**
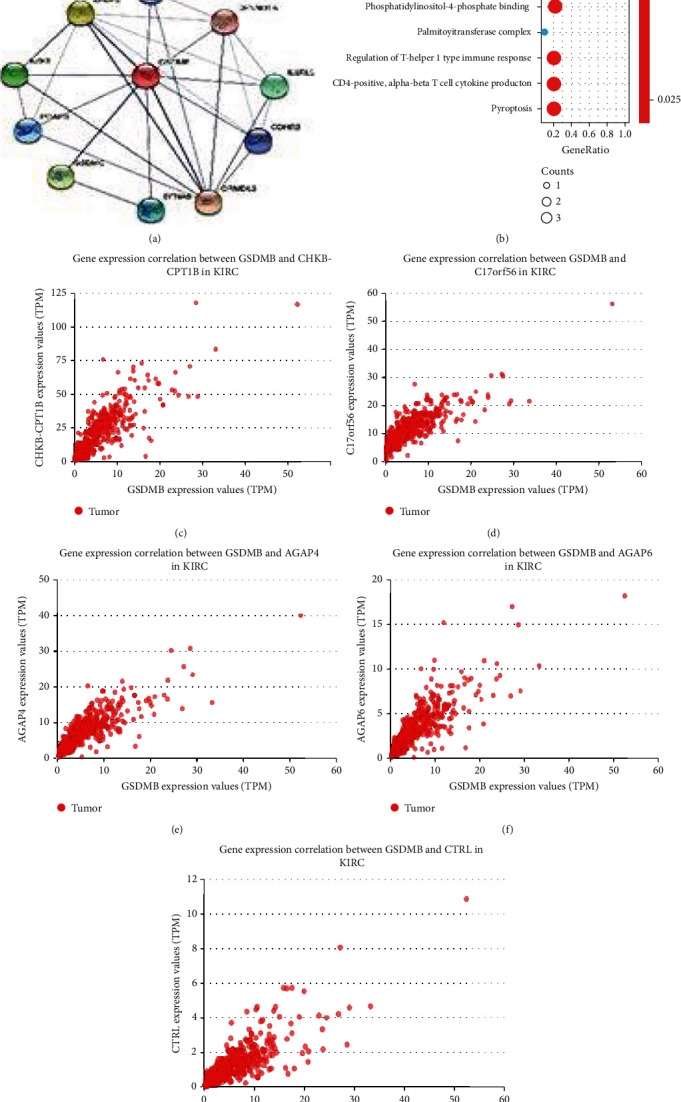
PPI networks and functional enrichment analyses. (a) A network of GSDMB and its coexpression genes. (b) Functional enrichment analyses of 10 involved genes. GSDMB was associated with cytokine-cytokine receptor interaction. (c–g) The correlation analyses between the expressions of GSDMB and coexpressed genes in ccRCC. ccRCC: clear cell renal cell carcinoma.

In order to build PPI networks and functional annotations, the STRING database, GO, and KEGG analyses were conducted. A network of GSDMB and its associated 10 coexpression genes is presented in Figure 7(a). Moreover, Figure 7(b) shows that the alterations in the biological process of GSDMB were related to cytokine-cytokine receptor interaction. Functional annotations have shown that these types of genes were most likely associated with the palmitoyltransferase complex. Figures 7(c)–7(g) show the correlation analyses between GSDMB expression and coexpressed genes in ccRCC from TCGA.

### 3.10. Correlation Analysis of GSDMB Expression and Immune Cell Infiltration in ccRCC

**Figure 8:**
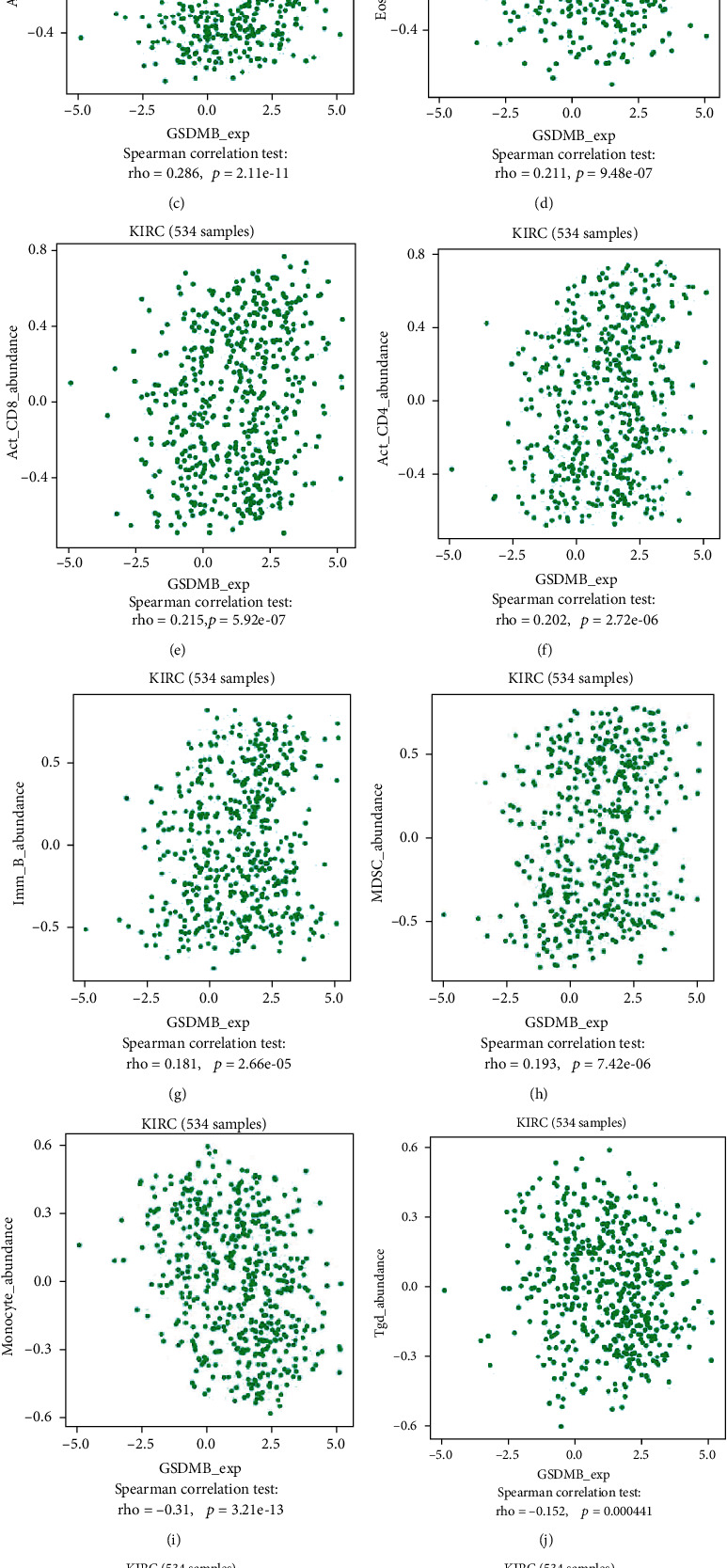
Correlations of GSDMB expression with immune infiltration level. (a) GSDMB expression is negatively related to tumor purity and has correlations with the B cell, CD8+T cell, CD4+T cell, macrophage, neutrophil, and dendritic cell in ccRCC. (b) Relations between the expression of GSDMB and 28 types of TILs across human cancers. (c–m) GSDMB was correlated with the abundance of activated B cells, eosinophils, activated CD8 T cells, activated CD4 T cells, immature B cells, myeloid-derived suppressor cells, monocyte cells, Tgd cells, NK cells, Th17 cells, and Treg cells. ccRCC: clear cell renal cell carcinoma.

The potential relation between the expression of GSDMB and the six different types of tumor-infiltrating immune cells was analyzed via the TIMER database.  Figure 8(a) shows that GSDMB expression was correlated with CD4+ T cells (*r* = 0.291, *P* = 1.90*e* − 10) and neutrophils (*r* = 0.144, *P* = 2.04*e* − 03). The relation between GSDMB expression and 28 different kinds of TILs as identified in the TISIDB database was also evaluated.  Figure 8(b) shows the associations between the expression of GSDMB and the 28 different types of TILs throughout human cancers. Figures 8(c)–8(m) show that GSDMB expression was associated with an abundance of activated B cells (*r* = 0.286, *P* = 2.11*e* − 11), eosinophil (*r* = 0.211, *P* = 9.48*e* − 7), activated CD8 T cells (*r* = 0.215, *P* = 5.92*e* − 7), activated CD4 T cells (*r* = 0.202, *P* = 2.72*e* − 6), immature B cells (*r* = 0.181, *P* = 2.66*e* − 5), myeloid-derived suppressor cells (MDSC) (*r* = 0.193, *P* = 7.42*e* − 5), monocyte cells (*r* = −0.31, *P* = 3.21*e* − 13), gamma delta T cells (Tgd) (*r* = −0.152, *P* = 0.000441), natural killer cells (NK) (*r* = −0.147, *P* = 0.000652), type 17 T helper cells (Th17) (*r* = 0.135, *P* = 0.00172), and regulatory T cells (Treg) (*r* = −0.109, *P* = 0.0114). These findings demonstrated that GSDMB may have a distinct function in immune infiltration in ccRCC.

### 3.11. Relationship between GSDMB and PD1/PD-L1 in ccRCC

**Figure 9:**
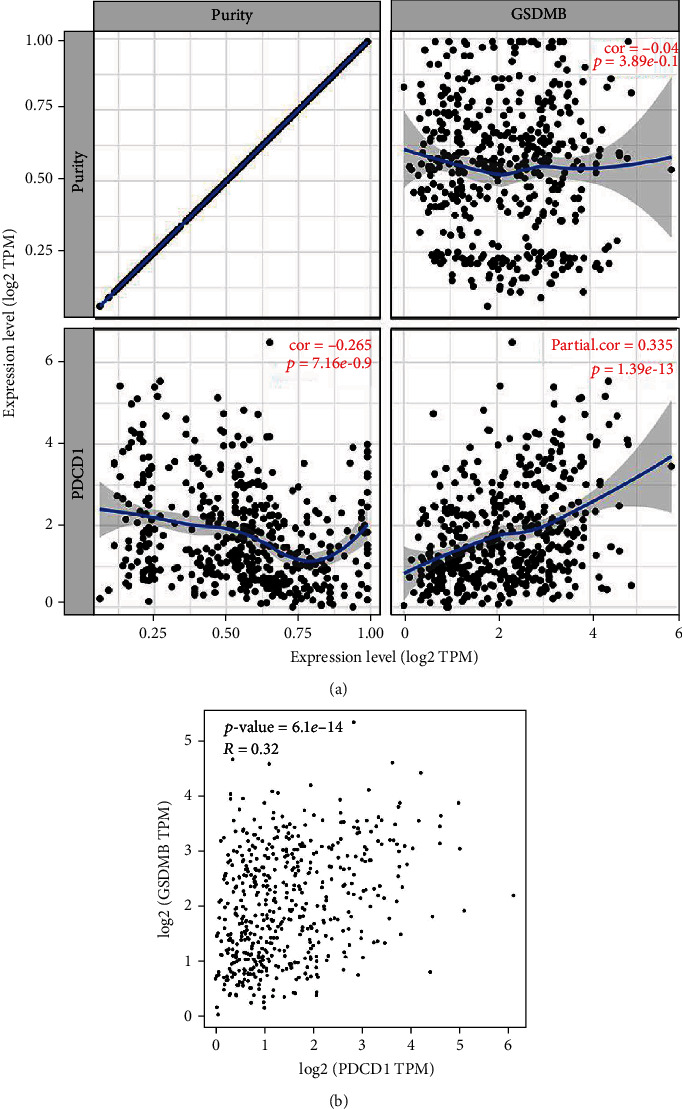
Relationship between GSDMB and PD1 in ccRCC. (a) Spearman correlation of GSDMB with expression of PD-1 in ccRCC adjusted by purity using TIMER. (b) The expression correlation of GSDMB with PD-1 in ccRCC determined by the GEPIA database. ccRCC: clear cell renal cell carcinoma.

Considering the potential oncogenic role of GSDMB in ccRCC, the relationship of GSDMB with PD1 or PD-L1 was assessed. As suggested in Figures 9(a) and 9(b), GSDMB expression was significantly positively correlated with PD1 in ccRCC. These results demonstrate that tumor immune escape might be involved in GSDMB-mediated carcinogenesis of ccRCC.

## 4. Discussion

In this article, we first revealed that the mRNA expression of GSDMB was not normal in different types of cancers. Then, we demonstrated that both mRNA and protein expressions of GSDMB were upregulated in ccRCC. Upregulated mRNA expression of GSDMB was related to a high T stage as well as high pathologic stage in a positive way. ROC curve analysis suggested that GSDMB may be a potentially valuable diagnostic biomarker for the differentiation between ccRCC and normal tissues. The results of the Kaplan-Meier curves and univariate analysis have demonstrated that high mRNA expression of GSDMB is correlated with short OS and DSS. Taken together, GSDMB could be valuable as a potential biomarker that is related to a poor and unfavorable prognosis in ccRCC. The nomogram was generated by integrating the clinical characteristics that were identified via multivariate analysis as being independently correlated with survival to present a quantitative model to clinicians, which can be helpful in predicting the prognosis of ccRCC patients. Besides, PPI networks and functional annotations we constructed. Moreover, GSDMB may have a distinct function in immune infiltration in ccRCC.

GSDMB was formerly referred to as GSDML (gasdermin-like protein). It is based in chromosome 17q21, where other genes that affect illnesses related to atypical immune responses might also be harbored. What is more, 17q21 also contains ORMDL3, which also has the ability to regulate GSDMB's expression [14]. Recent studies have shown that GSDMB is capable of inducing pyroptosis-like features; however, it is still unknown if GSDMB can generate pyroptosis or in what way GSDMB mechanistically takes part in this inflammatory regulation [15]. The N-terminal domain of GSDMB could link up with sulfatide distinctively, and since the overexpression of sulfatide is often associated with the progression of cancer, it suggests that GSDMB may have a significant function in cancer cell metastasis and migration [16–19]. A few articles regarding the role of GSDMB in oncogeny of few different cancers have been published recently, involving breast cancer, gastric cancer, and cervical squamous cell carcinomas [20–22]. Nevertheless, a comprehensive exploration of GSDMB's expression and its value as a prognostic indicator in ccRCC has not been performed. In our research, according to the pan-cancer analysis, we demonstrated that GSDMB mRNA is atypically expressed in different types of cancers. Furthermore, we certified that GSDMB was significantly upregulated in ccRCC.

So far, the specific role of GSDMB in tumors has not been reported comprehensively. The previous article suggests that the inhibition of Hsp90 may be a new mechanism that could block GSDMB-2 and prevent it from applying its tumorigenic potential [23]. Other studies demonstrated that the expression levels of GSDMB and Alu versus long-terminal region- (LTR-) derived promoter utilization could be valuable markers in assessing the growth and development of gastric cancer [24, 25]. Lutkowska et al. have proven that polymorphisms of two nonmajor histocompatibility loci, in squamous cell carcinomas, are related to invasive cervical cancer [26]. One of these identified polymorphisms is the single-nucleotide polymorphism NC_000017.10: g.38051348A>G (rs8067378), based in 9.5 kb downstream from the location of GSDMB. This is equivalent to the LTR and the cellular promoter, which could prompt GSDMB expression. In this article, the results of the coexpression analyses have shown that GSDMB expression is significantly associated with that of the palmitoyltransferase complex, while this should be tested by other experiments. All of the results above indicate that GSDMB could be a potential valuable biomarker or possible target in cancer treatment. To verify the clinical value of GSDMB in diagnosing ccRCC, a ROC curve analysis was conducted. Our findings demonstrated that GSDMB had a significantly greater AUC value in the identification of ccRCC. In addition, the results of the Kaplan-Meier curves and log-rank test have shown that ccRCC patients with a high level of GSDMB mRNA expression are related to a reduced OS and DSS compared to patients with low levels of GSDMB. Based on these findings, we conclude that GSDMB may function as a prospective diagnostic biomarker that can be of value in the differentiation between ccRCC and normal tissues.

The GSDM family has roles in the management of cell differentiation and proliferation, particularly in the process of pyroptosis. Pyroptosis is a new kind of programmed cell death that has vital functions in immune defenses [27]. In 1992, it was observed for the first time in macrophages, which were infected by the Gram-negative bacteria Shigella flexneri; however, this term only became known after 2001 when it was referred to as such by Lawrence H. Boise [28]. Pyroptosis arises via the activity of different stimuli and inflammatory caspases which influence cleavage of the GSDM family and the discharge of its N-terminal effector domain as well as C-terminal inhibitory domain [29]. The N-terminal domain oligomerizes the inside of the membrane of the cell and creates pores, resulting in the quick rupture of plasma membranes, thereby discharging the contents in the cell and proinflammatory mediators like interleukin- (IL-) 1*β* and IL-18 [30]. The discharge of molecular patterns associated with damage from lysed pyroptotic cells can lead to the recruitment of immune cells and increases to stimulate inflammation. Studies demonstrated that GSDMB is involved in pyroptosis: cleavage of the GSDMB protein by caspase-1 causes pyroptosis [31], GSDMB stimulates noncanonical pyroptosis through increasing the activity of caspase-4 [32], and caspase-3/-6/-7 can cleave GSDMB [15]. Nonetheless, the correlation analysis of GSDMB expression and immune cell infiltration in ccRCC has not been studied. Our study has shown that multiple immune cells that infiltrate tumors (CD4-positive T cells and neutrophils) were associated with GSDMB expression in ccRCC through using TIMER. In addition, we also demonstrated that a positive relation was observed among GSDMB expression and abundance of activated B cells, eosinophils, activated CD8 T cells, activated CD4 T cells, immature B cells, MDSC, monocyte cells, Tgd cells, NK cells, Th17 cells, and Treg cells. These outcomes indicate that a potential association exists between GSDMB and immune infiltration in ccRCC. Besides, relationship between GSDMB and PD1/PD-L1 in ccRCC was explored. We found that GSDMB expression was significantly positively correlated with PD1 in ccRCC. It demonstrates that tumor immune escape might be involved in GSDMB-mediated carcinogenesis of ccRCC. Nonetheless, continuing research should be conducted to further verify this association.

A few limitations exist in the present article. Firstly, GSDMB's expression and its prognostic significance were investigated with publicly available online databases; more research in which clinical samples are analyzed is needed to verify the above findings. Besides, to provide additional support on the precise process of how GSDMB impacts immune infiltration in ccRCC, in vivo/vitro experiments need to be performed.

## 5. Conclusions

Conclusively, in the present study, we have shown that mRNA and protein expression of GSDMB is upregulated in ccRCC and associated with a high TNM stage in a positive way for the first time. This study indicates that GSDMB may be recognized as a potential biomarker associated with poor prognosis, which can be used to detect ccRCC patients that have clinical outcomes which are poor and could have a distinct function in immune infiltration.

## Figures and Tables

**Table 1:** The clinical characteristics of ccRCC patients between the high GSDMB expression group and low expression group.

Characteristic	Low expression of GSDMB	High expression of GSDMB	*P*
*n*	269	270	
T stage, *n* (%)			0.040
T1	154 (28.6%)	124 (23%)	
T2	33 (6.1%)	38 (7.1%)	
T3	79 (14.7%)	100 (18.6%)	
T4	3 (0.6%)	8 (1.5%)	
N stage, *n* (%)			0.593
N0	115 (44.7%)	126 (49%)	
N1	6 (2.3%)	10 (3.9%)	
M stage, *n* (%)			0.358
M0	225 (44.5%)	203 (40.1%)	
M1	36 (7.1%)	42 (8.3%)	
Pathologic stage, *n* (%)			0.093
Stage I	150 (28%)	122 (22.8%)	
Stage II	28 (5.2%)	31 (5.8%)	
Stage III	52 (9.7%)	71 (13.2%)	
Stage IV	38 (7.1%)	44 (8.2%)	
Primary therapy outcome, *n* (%)			0.861
PD	5 (3.4%)	6 (4.1%)	
SD	4 (2.7%)	2 (1.4%)	
PR	1 (0.7%)	1 (0.7%)	
CR	76 (51.7%)	52 (35.4%)	
Gender, *n* (%)			0.433
Female	88 (16.3%)	98 (18.2%)	
Male	181 (33.6%)	172 (31.9%)	
Race, *n* (%)			0.057
Asian	1 (0.2%)	7 (1.3%)	
Black or African American	25 (4.7%)	32 (6%)	
White	239 (44.9%)	228 (42.9%)	
Age, *n* (%)			0.064
≤60	123 (22.8%)	146 (27.1%)	
>60	146 (27.1%)	124 (23%)	
Histologic grade, *n* (%)			0.324
G1	6 (1.1%)	8 (1.5%)	
G2	126 (23.7%)	109 (20.5%)	
G3	99 (18.6%)	108 (20.3%)	
G4	32 (6%)	43 (8.1%)	
Serum calcium, *n* (%)			0.446
Elevated	3 (0.8%)	7 (1.9%)	
Low	104 (28.4%)	99 (27%)	
Normal	75 (20.5%)	78 (21.3%)	
Hemoglobin, *n* (%)			0.073
Elevated	2 (0.4%)	3 (0.7%)	
Low	125 (27.2%)	138 (30.1%)	
Normal	111 (24.2%)	80 (17.4%)	
Laterality, *n* (%)			0.012
Left	111 (20.6%)	141 (26.2%)	
Right	158 (29.4%)	128 (23.8%)	
OS event, *n* (%)			<0.001
Alive	203 (37.7%)	163 (30.2%)	
Dead	66 (12.2%)	107 (19.9%)	
DSS event, *n* (%)			0.006
Alive	224 (42.4%)	196 (37.1%)	
Dead	41 (7.8%)	67 (12.7%)	
PFI event, *n* (%)			0.140
Alive	197 (36.5%)	181 (33.6%)	
Dead	72 (13.4%)	89 (16.5%)	
Age, median (IQR)	61 (53, 70)	60 (51, 69)	0.351

ccRCC: clear cell renal cell carcinoma.

**Table 2:** Univariate and multivariate Cox proportional hazards analyses of MGSDMB expression and OS for patients with ccRCC.

Characteristics	Total (*N*)	Univariate analysis		Multivariate analysis	
		Hazard ratio (95% CI)	*P* value	Hazard ratio (95% CI)	*P* value
T stage	539				
T1 & T2	349	Reference			
T3 & T4	190	3.228 (2.382-4.374)	<0.001	1.373 (0.605-3.117)	0.448
N stage	257				
N0	241	Reference			
N1	16	3.453 (1.832-6.508)	<0.001	1.426 (0.708-2.872)	0.321
M stage	506				
M0	428	Reference			
M1	78	4.389 (3.212-5.999)	<0.001	2.494 (1.475-4.217)	<0.001
Pathologic stage	536				
Stage I & stage II	331	Reference			
Stage III & stage IV	205	3.946 (2.872-5.423)	<0.001	1.468 (0.579-3.725)	0.419
Gender	539				
Female	186	Reference			
Male	353	0.930 (0.682-1.268)	0.648		
Age	539				
≤60	269	Reference			
>60	270	1.765 (1.298-2.398)	<0.001	1.766 (1.152-2.707)	0.009
Histologic grade	531				
G1 & G2	249	Reference			
G3 & G4	282	2.702 (1.918-3.807)	<0.001	1.666 (1.003-2.768)	0.049
GSDMB	539				
Low	270	Reference			
High	269	1.957 (1.434-2.671)	<0.001	2.059 (1.303-3.255)	0.002

ccRCC: clear cell renal cell carcinoma.

## Data Availability

The data presented in this study are available within the article materials.
